# Diabetes and natural and man-made disasters: prevention, preparation, response and recovery

**DOI:** 10.1007/s00125-025-06406-6

**Published:** 2025-04-15

**Authors:** Andrew J. M. Boulton, Alicia J. Jenkins, Brij Makkar, Boris Mankovsky, Merhawit A. Abera, Solomon Tesfaye

**Affiliations:** 1 Department of Diabetes, Endocrinology and Gastroenterology, School of Medical Sciences, University of Manchester, Manchester, UK; 2 Baker Heart and Diabetes Institute, Melbourne, VIC Australia; 3 Dr Makkar’s Diabetes & Obesity Centre, New Delhi, India; 4 Department of Diabetology, National University of Healthcare, Kyiv, Ukraine; 5 Mekelle University College of Health Sciences, Mekelle, Ethiopia; 6 Diabetes Research Unit, Sheffield Teaching Hospitals NHS Foundation Trust, Sheffield, UK

**Keywords:** COVID-19 pandemic, Diabetes, Guidelines, Man-made disasters, Natural disasters, Review

## Abstract

Both the global prevalence of diabetes and the frequency of natural and man-made disasters are increasing. Of all chronic diseases, the consequences of sudden loss of medical supplies are most serious for those with diabetes, with people living with type 1 diabetes being at risk of death within a few days without insulin. This review considers how to prepare for and respond to sudden reductions in medical supplies to those with diabetes. Recent experiences with the COVID-19 pandemic in India, the war in Ukraine and the war/blockade in the Tigray region of Ethiopia are described, and the importance of prevention, preparedness, response and recovery are discussed. It is hoped that lessons from these and other disasters and ongoing advocacy and other actions may help to mitigate the risks of significant morbidity and mortality for people with diabetes in disaster-impacted regions across the world.

**Graphical Abstract:**

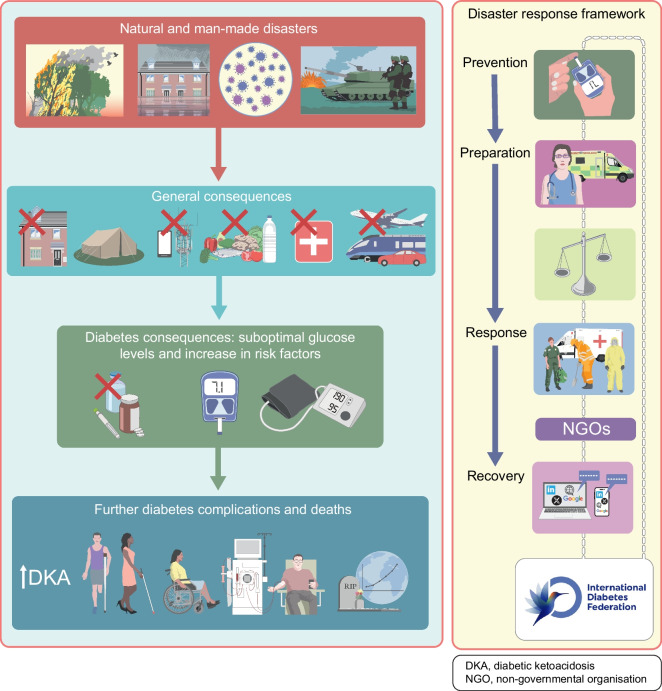

**Supplementary Information:**

The online version contains a slideset of the figures for download available at 10.1007/s00125-025-06406-6.

## Introduction

The word ‘disaster’ is derived from the French word ‘desastre’ meaning a bad or evil star. A disaster may also be defined as a serious disruption to community life that threatens or causes death or injury and/or property damage beyond the day-to-day capacity of local authorities and which requires special resources other than those that are normally available [1]. Disasters may be either natural or man-made. Examples of natural disasters include hurricanes, typhoons, earthquakes, tsunamis and bushfires (wildfires). Man-made disasters, which may result from human intent, negligence or error, include nuclear catastrophes such as Chernobyl, war, blockades, civil disorder and terrorism.

There has been a recent global increase in disasters, which has been accompanied by a rapid rise in the global prevalence of diabetes, with over half a billion adults living with diabetes in 2021: the increase recorded between 2019 and 2021 was 16% according to the International Diabetes Federation (IDF) Diabetes Atlas [2]. Insulin is one of the few drugs that, if acutely unavailable, can result in death within days in people with type 1 diabetes. Moreover, those with type 2 diabetes, who are frequently on multiple medications including insulin or other injectables, are also at great risk if supply chains are disrupted. In any disaster, natural or man-made, it is essential to support all people with diabetes, irrespective of their age, gender, race and ethnicity, country of origin or residence, religion or politics, education or socioeconomic status, or which ‘side’ they are on. Even in times of war the rights to food, shelter and medical care are basic human rights [3, 4]. Unfortunately, these key human rights cannot always be met, even in times of peace, enhancing the need for awareness, advocacy, collaboration and change [4]. A country’s capacity, particularly if less advantaged, to provide for its population can be reduced for some time after a major disaster, such as a tsunami, pandemic or war, lessening its ability to cope with a subsequent disaster.

Sadly, therefore, the need to review diabetes management in disasters, including preparedness, is crucial. This review focuses on diabetes care in natural and man-made disasters. First, the importance of vulnerability, access to care, supply chains and future planning is discussed. Subsequent sections focus on natural disasters, using the example of the COVID-19 pandemic, and man-made disasters, using examples of the war in Ukraine and the war and civil unrest in the Tigray region of Ethiopia. Finally, the role of the IDF in disaster management is briefly discussed.

## General consequences of a disaster

A disaster may lead to loss of shelter, power, clean water and a secure healthy food supply. Communication systems, including telephones and the internet, which are vital in disaster responses, may be lost, so paper back-up of key information is crucial, as are alternative means of communication such as ham radios and satellite phones. People may need to evacuate, sometimes to another country or to a refugee camp or, perhaps, as seen during the COVID-19 pandemic, may need to shelter in place for prolonged periods of time. In shelters and evacuation centres food and water supplies, healthcare facilities, sanitation and space may be suboptimal. In addition, some people with diabetes may be previously undiagnosed or not well educated in their self-care, or prefer not to declare their diabetes status, and staff may not know when, or have the means, to contact suitably qualified healthcare professionals [5]. Similar issues often arise in refugee camps, where people may be housed for many years, including those with impaired glucose tolerance or at risk of diabetes or with undiagnosed diabetes. Healthcare services in refugee camps should screen for diabetes and healthcare professionals should be well trained and equipped to provide diabetes care. For example, the United Nations Relief and Works Agency (UNRWA), which provides care for Palestinian refugees in the Middle East, introduced diabetes and hypertension care programmes in 1992 that include community health workers [6–8].

Medicines and related devices and medical records, diagnostic and treatment facilities (e.g. hospitals, dialysis units, clinics and eye screening programmes) and related staff may be lost or diverted during disasters, and available healthcare teams may not be knowledgeable in diabetes care, nor have access to adequate diabetes-related medicines and devices. Even if a disaster is unrelated to infection, infections may arise during a disaster or afterwards as a result of contaminated water or food supplies, people living in close proximity or injuries [5]. Reserve medical supplies may not be adequate or accessible, supply and distribution chains may be disrupted, and emergency aid shipments may be denied clearance. During and after a disaster, metabolic control and risk factors (e.g. blood pressure) usually worsen, increasing the risk of new-onset prediabetes and type 2 diabetes and of chronic diabetes complications. Psychological stress can also be severe and prolonged. In addition, stress can affect healthcare and aid workers, so attention should also be given to their care. Disaster-related challenges are summarised in the Text box, ‘Consequences of a disaster’.


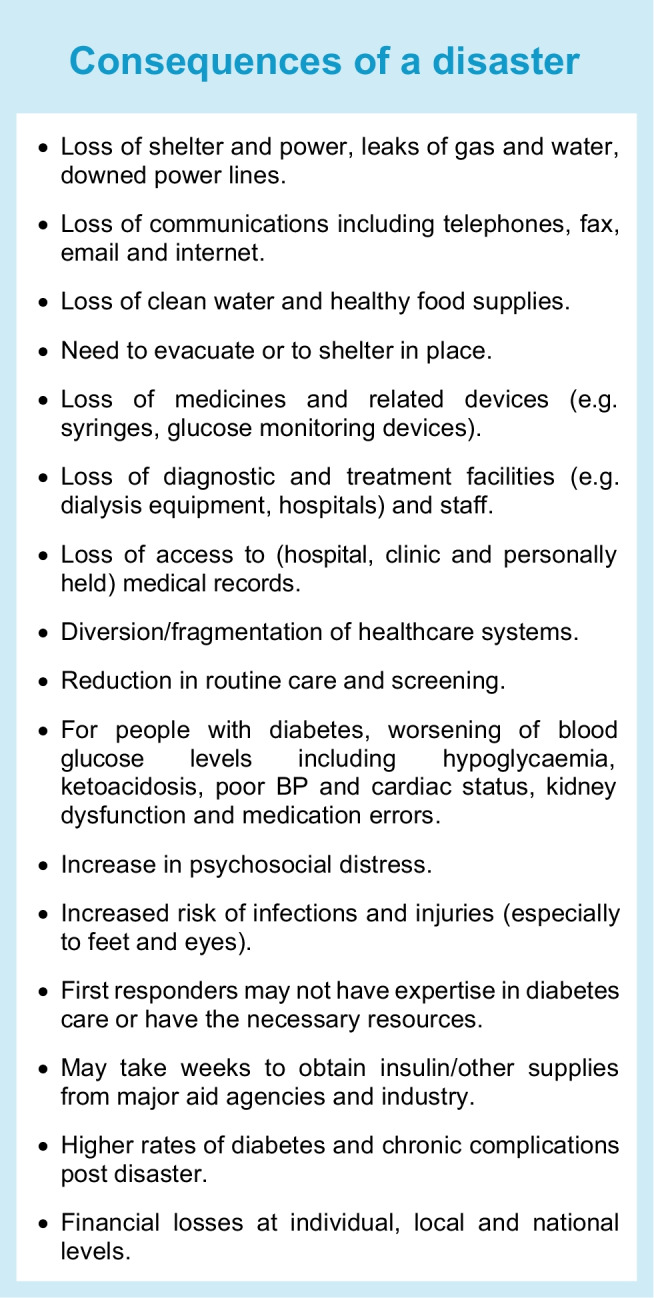


### Disaster prevention, preparedness, response and recovery framework

Injury, death and damage in disasters can be reduced through careful forward planning, preparation and responses. Many individuals and groups can assist in disaster prevention, preparation, response and recovery, ranging from individuals with diabetes and their families to clinicians and the healthcare system, diabetes associations (professional and lay), local and national governments and local, national and international non-governmental organisations. Local input is essential, especially if aid is to come from overseas, for example local assistance with the receipt and distribution of aid. Local knowledge of geography, languages and needs, as well as relevant, ideally prearranged, contacts, is key to successful disaster management and should be outlined in disaster preparation plans. The Text box, ‘Contacts and resources that can assist in disaster prevention, preparation, response and recovery’, lists groups that medical system representatives may need to interact with during a disaster.


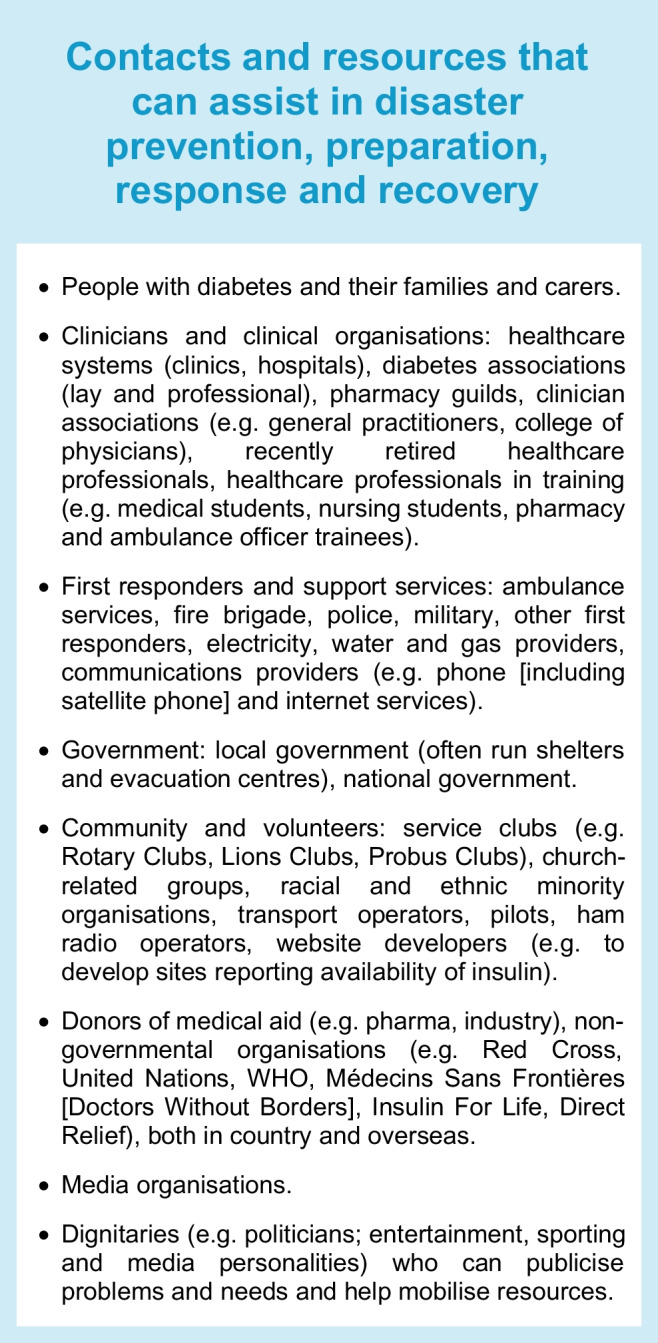


Learning from past experiences is helpful and planning ahead for future disasters is important, as is the sharing of any relevant information. Mortality rates from disasters are declining in some countries, probably because of better preparedness, although this is not the case in disadvantaged countries [9]. In a systematic review of 1992–2018 publications on diabetes care during natural and man-made disasters in low- and middle-income countries, Kehlenbrink et al identified that the burden of diabetes was not adequately captured nor addressed and clinical guidance was insufficient, with a need for simple cost-effective models of diabetes care delivery during disasters [10]. Some less advantaged countries, specifically Iran, have shared their approach to and findings related to the disaster-preparedness of their hospitals (e.g. [11]).

In man-made disasters such as wars and civil unrest, some parties in a conflict may blockade essential medical aid and food supplies [4]. Multi-faceted means to promptly and effectively overcome such inhumane acts must be identified and activated, as in the Ethiopian conflict (see ‘The Tigray war’). As Martin Luther King Jr said, ‘In the end, we will remember not the words of our enemies, but the silence of our friends’. Let us not be silent friends. One should think ‘Disasters and diabetes—not if, but when’, and ‘How can I help?’

**Fig. 1:**
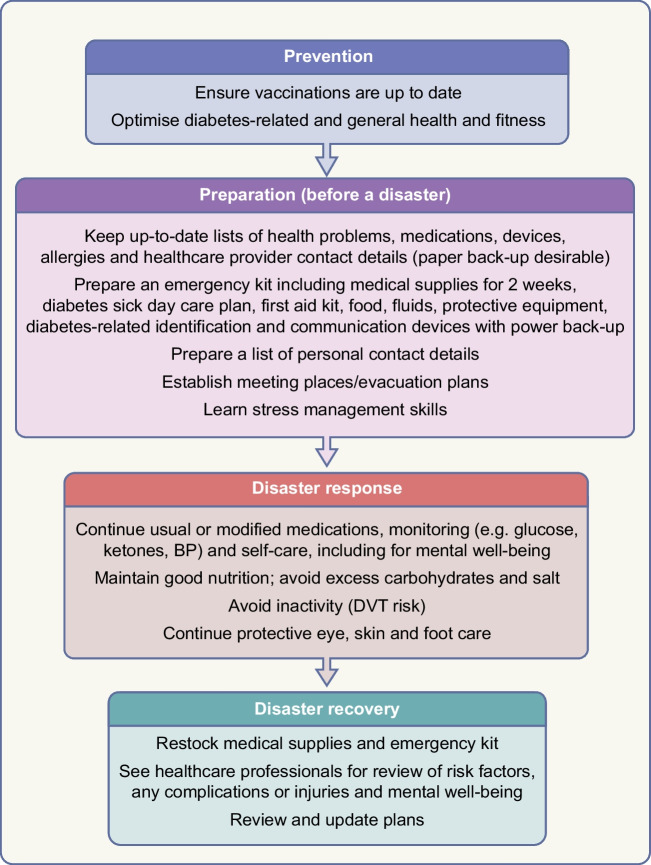
Overview of disaster-related actions recommended for people with diabetes and their families according to the prevention, preparation, response and recovery approach. DVT, deep vein thrombosis. This figure is available as part of a downloadable slideset

**Fig. 2:**
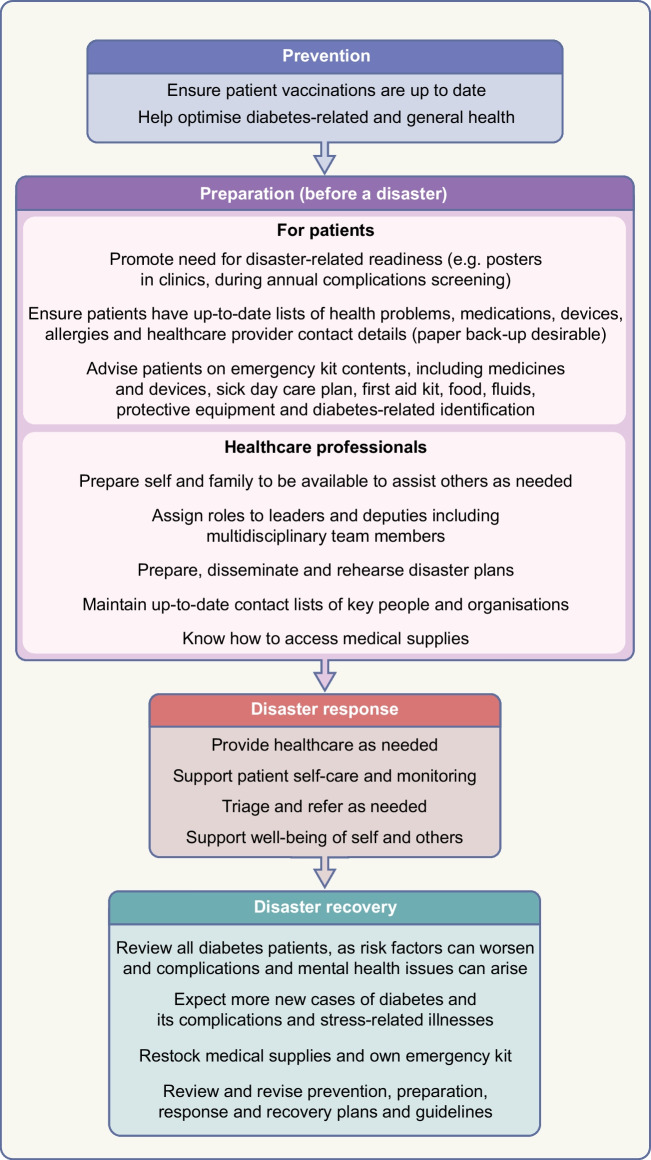
Overview of disaster-related actions recommended for healthcare professionals according to the prevention, preparation, response and recovery approach. This figure is available as part of a downloadable slideset

**Fig. 3:**
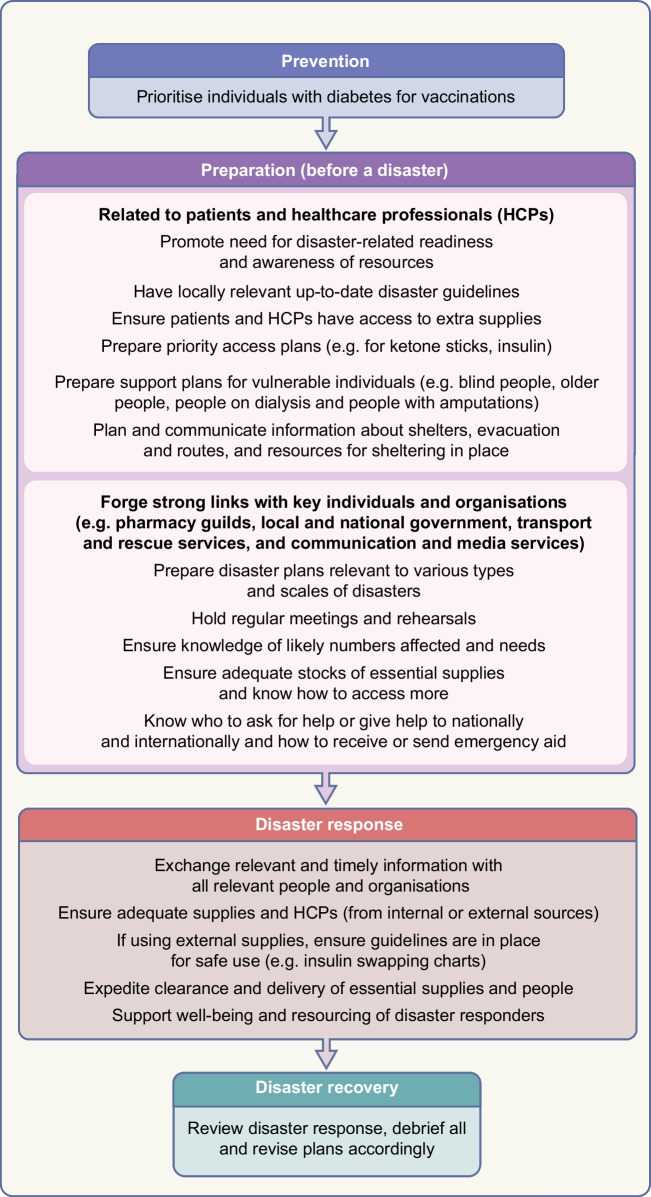
Overview of disaster-related actions recommended for diabetes associations and governments according to the prevention, preparation, response and recovery approach. This figure is available as part of a downloadable slideset

A robust disaster management plan, including key contact details and ideally in place and regularly updated and rehearsed well before a disaster, can mitigate adverse outcomes of disasters. The classic approach to disaster management is that of prevention where possible (such as by vaccination against infectious agents), preparation, response and recovery. Ideally, disaster guidelines should consider local ways of living, languages, challenges and support resources, and should be relevant to various types of disasters, particularly those more likely to separate a person from their medical supplies and those in which (as in the COVID-19 pandemic) prolonged sheltering in place is required. Disaster plans are particularly important for high-risk people such as those with type 1 diabetes, pregnant women, young people, older people and people with chronic complications or comorbidities. For example, people on dialysis will need special consideration, which may include early evacuation [12]. In all disasters, multiple stakeholders, from individuals to families and local communities, at national and international levels, may be involved. Access to adequate essential medical supplies and healthcare is essential, which may require external assistance. Aid is usually available from many sources, from rapid early responders to large-scale long-term assistance, but internal coordination and local knowledge are essential to enable the aid and related knowledge to get to those in need in a timely manner. Preparedness is key at multiple levels, yet is often suboptimal, costing time and even lives during a disaster. While the IDF, some national diabetes organisations and other organisations have published diabetes-related disaster guidelines [12–16], many diabetes organisations have not. Figures 1, 2 and 3, updated from principles outlined in the IDF Western Pacific Region (IDF-WPR) guidelines on diabetes care and disasters [13, 14], discussed further later in this review, provide overviews of disaster-related roles recommended for individuals and their families, healthcare professionals and diabetes associations and governments, respectively, following the prevention, preparation, response and recovery approach. Key elements are knowing and sharing where diabetes supplies are available and when and how to obtain healthcare professional input. Identifying contacts and roles pre disaster is highly recommended. Most countries have a disaster response plan and committee(s), but are not aware of diabetes-related needs. Diabetes associations should help fill this void.

The following sections discuss natural and man-made disasters and their implications for people with diabetes, including examples and resources.

## Natural disasters

Natural disaster rates are increasing globally [9], most likely due to climate change and the emergence of novel infectious diseases. Apart from that related to the COVID-19 pandemic, in recent decades there have been substantial reductions in disaster-related death rates, and probably also morbidity, owing to better standards of living and more robust infrastructure and disaster response systems. However, people in lower income regions still fare worse during disasters, with recent ratios of deaths between low- and high-income regions ranging manyfold [9]. The following section discusses the COVID-19 pandemic, using the example of India as a case study.

### COVID-19 pandemic

People with diabetes are at increased risk of infections and more adverse outcomes than people without diabetes, as was seen during the COVID-19 pandemic. A pandemic was declared by the WHO in February 2020 and outbreaks are still ongoing, with mitigation due to isolation, good hygiene practices including the wearing of masks and, where available, the use of vaccines and more recently antiviral agents. Not unexpectedly, it was recognised early in the pandemic that people with diabetes, especially those with obesity, hypertension, heart disease, chronic kidney disease and metabolic dysfunction-associated steatotic liver disease, were at increased risk of severe COVID-19 requiring hospitalisation and ventilatory support and at increased risk of death [17–21]. Adverse outcomes, including mortality, were more frequent with older age and in women, racial and ethnic minority groups and lower socioeconomic groups. Subsequent studies revealed a COVID-19-related increase in type 2 diabetes and in new-onset type 1 diabetes (perhaps related to direct beta cell toxicity), mainly in youth, who often presented in diabetic ketoacidosis [22–24]. As with other (natural or man-made) disasters, COVID-19 is associated with worsening of metabolic control and traditional risk factors for diabetes (e.g. adiposity, hypertension and dyslipidaemia), lower levels of physical activity, increased psychological stress, reduced access to usual medical care and increased rates of chronic diabetes complications, including vision loss and heart disease [25]. However, data on increased rates of renal disease and amputations are equivocal [25].

COVID-19 has challenged individuals, healthcare systems, governments and economies globally, affecting the livelihoods of many individuals, but has also provided valuable lessons on how to deal with disasters. Some advances, such as the increased use of telemedicine and self-monitoring and development of related skills, continue to benefit clinical practice and people with diabetes, even after the initial acute event [3].

A series of articles including treatment strategies for people with COVID-19 and diabetes and a plan for healthcare system recovery from the COVID-19 pandemic have been published [20, 26, 27]. During the pandemic, the lockdowns, public health measures, diversion of healthcare resources and funds to deal with its direct consequences, loss of many healthcare professionals and supply chain issues negatively affected routine diabetes care, reduced self-care capacity and delayed new diagnoses [20, 27]. Comparisons with pre-pandemic metrics showed that even a 12 month delay in routine care worsened risk factors, chronic complications and mortality risk in people with diabetes [20]. As disasters usually have a greater negative impact on high-risk groups and in lower socioeconomic settings, often aggravating health inequalities, it is recommended that, in the recovery phase, ambulatory diabetes services be prioritised towards those at high risk of adverse outcomes to improve health outcomes and reduce inequity [20, 26, 27]. In the remainder of this section the experience of India in relation to diabetes and the COVID-19 pandemic is discussed. Other types of disasters have similar consequences.

### Case study: India

India is a multiethnic country with >1.4 billion people, including >101 million people living with diabetes [2, 28, 29]. The country is divided into 36 regions, with two-thirds of people living in rural areas, and there are high levels of illiteracy, making a uniform approach to disaster management difficult. By mid-May 2021, India had nearly 25 million COVID-19 cases and >270,000 recorded deaths (approx. 1.1% of the number of cases) [30]. As described above and in the Text box, ‘Consequences of a disaster’, during the pandemic, people in India faced relocation, confinement to their homes, isolation, reduced opportunities to perform physical activity, loss of income and lack of healthy food; in addition, those with diabetes also faced limited supplies and often a complete lack of diabetes-related medicines and equipment, including insulin and glucose monitoring equipment, and a reduction in follow-up care [31]. While many people lost their jobs, those in the healthcare professions faced greater workloads. Women in India seemed to be particularly badly affected, potentially because of the increase in domestic workloads [32]. Other consequences noted, as previously reported in relationship to a 2004 tsunami [33], included increases in domestic violence and stress eating, higher levels of psychological distress and a higher prevalence of diabetes and impaired glucose tolerance in the affected population. In addition, as in many other countries, people hospitalised with COVID-19 had a high prevalence of diabetes, more severe disease, higher rates of adverse events and a higher mortality risk [34, 35]. In India, COVID-19 was also associated with significantly higher rates of new-onset diabetes [35], suboptimal glucose management and the need for hospitalisation in those with pre-existing diabetes [36]. Being admitted to hospital was more difficult during the pandemic, as most hospitals were dedicated to COVID-19 care only; hence, many diseases, especially chronic diseases, were neglected. While infections are common during disasters, an unusual observation during the pandemic was the high rate of mucormycosis reported in people with COVID-19 and diabetes, especially from India. Of the 101 mucormycosis cases reported up to May 2021, 82 (81%) were from India and 19 (19%) were from the rest of the world. Contributory factors were likely to be the marked hyperglycaemia and rampant use of corticosteroids in people with COVID-19 [37].

The government of India acted rapidly in tackling COVID-19 by the timely dissemination of information, creating more healthcare facilities and recruiting healthcare professionals to take care of the large numbers of affected people, creating digital solutions to track and monitor affected individuals and rapidly developing and implementing a mass vaccination programme. Use of technology, digital resources and communications were crucial, enabling efficient dissemination of awareness about COVID-19 to the public and updating of clinicians regarding the management of diabetes and COVID-19 (including through the use of emails, WhatsApp messages and Zoom webinars from the Research Society for the Study of Diabetes in India [RSSDI]). Although acceptance of telemedicine is suboptimal in India, greater accessibility and acceptance of technology during the pandemic helped individuals with diabetes to connect with their clinicians and achieve better metabolic control [36, 38]. As in the IDF-WPR [39] and other parts of the world, tele/digital medicine consultations enabled people with diabetes to obtain medical advice, and the use of connected glucose meters helped keep them and their clinicians updated on progress. Insulin initiations could be performed using demonstration videos and during video consultations. Prescriptions could be sent to individuals with diabetes via digital platforms and medications/supplies could be sourced through online pharmacies, which were then delivered to individuals’ doorsteps, even during lockdowns. However, although technology use should feature in preparedness for and responses during future disasters, it must be recognised that the required resources, for example for communication or for healthcare, may not always be available, and other means will be required. Forward planning and resilience are key.

## Man-made disasters

Man-made and natural disasters have many elements in common regarding prevention, preparation, response and recovery and their consequences, affecting, in particular, vulnerable groups such as those with diabetes and major comorbidities, pregnant women, children and older people. During man-made disasters there may be risks of radiation, biological warfare and use of weapons of mass destruction. There may also be deliberate blockades of humanitarian aid, including essential medicines such as insulin. Two examples of man-made disasters, the wars in Ukraine and Ethiopia, are discussed in the following sections.

### Case study: the Russia–Ukraine war

Ukraine, the largest country in Central Europe, with a prewar population of approximately 42 million, had a diabetes prevalence in 2021 of about 7.1% among adults aged 20–79 years, representing about 2.3 million people [2], of whom about 200,000 required daily insulin injections, provided free by the state. In 2014, the Crimean Peninsula, part of Ukraine, was annexed by Russia and fighting between the two countries in two eastern regions of Ukraine began. These political and military events caused a significant migration of people, including individuals with diabetes and doctors, from affected areas to other parts of the country, mainly to regions nearby (including Kharkiv, Dnipro and Kyiv). The healthcare system adjusted and continued to operate at efficient levels. However, on 24 February 2022, a full-scale war started with the rapid movement of Russian military forces deep into Ukraine. The healthcare system and supply chains were not prepared and dramatic problems were evident from the very first hours and days of the war. A major challenge was the severe shortage of insulin, other glucose-lowering medications and diabetes devices (e.g. glucose meters and insulin pumps), as well as medications for treating other chronic conditions, for example l-thyroxine for treating hypothyroidism. Most people who needed insulin had enough supplies for only 2–3 weeks. About half of the pharmacies in the country closed because of a lack of supplies and staff, and delivery of insulin and other medications to areas affected by heavy fighting was severely limited and often impossible [40, 41]. During the first few weeks of the war, many people and medical personnel had to move. The chaos led to a very uneven geographical distribution of individuals with diabetes, with the number of people with diabetes in western parts of Ukraine increasing two- to threefold in the first few weeks of the war, which overwhelmed local facilities [42–44]. An estimated 11 million people had to leave their homes and >6 million people found asylum in other countries [45, 46]. However recent reports related to Ukrainian refugee children with type 1 diabetes in their host countries show good uptake of diabetes technology and relatively good metabolic control.

Ukraine received humanitarian aid from government and non-governmental organisations worldwide. With regard to diabetes, insulin delivery became the main priority early on and was mainly solved by the joint coordinated efforts of government, national and international non-governmental organisations and volunteers and donors. Pharmaceutical companies and non-governmental organisations (including many Insulin For Life affiliates [https://www.insulinforlife.org] and Direct Relief [https://www.directrelief.org]) provided insulin for free, which, as the traditional channels for supply of insulin had stopped, was successfully delivered by non-conventional carriers (supermarkets chains, postal workers and volunteers). Any co-payment for costly insulin analogues was immediately cancelled. A special order of the Ministry of Health, including a list of insulins approved for use, was prepared, and insulin swapping charts were made available, which were very helpful for clinicians who were not very familiar with diabetes management pre war. As many individuals with diabetes had to be treated by different healthcare providers from those normally treating them, the following actions were implemented:

- Doctors other than endocrinologists or general practitioners were able to write prescriptions for insulin and glucose-lowering medications.
- Webinars suitable for healthcare professionals who were relatively new to diabetes care commenced.
- Involvement of diabetes-related pharmaceutical company representatives was encouraged without commercial promotion to assist with information regarding the availability of their products and humanitarian aid.
- Collaboration with colleagues in European countries treating refugees with diabetes facilitated knowledge transfer.
- With the help of the European Society of Endocrinology, leaflets in Ukrainian including basic information on the management of hyper- and hypoglycaemia emergencies were prepared and were freely available for refugees at the borders.

Now in the fourth year of war, medical care in Ukraine, including diabetes care, has somewhat stabilised, and the supply of insulin and other glucose-lowering drugs has been restored to prewar levels. The migration of individuals with diabetes and medical personnel has also significantly reduced, even in areas close to the zones of fighting. For example, the number of people admitted to hospital with endocrine diseases (mainly diabetes) in the Kharkiv area was 410,203 in 2021, 294,489 in 2022 and 431,473 in 2023 (I. Smirnov, City Hospital, Kharkiv, Ukraine, personal communication, October 2024). Key lessons learnt to date from the war in Ukraine that can be applied to subsequent disasters are as follows: (1) people with insulin-treated diabetes need to have at least 2–3 months’ supply of diabetes medications; (2) non-conventional carriers can be used for insulin delivery; (3) partnerships with pharmaceutical companies are valuable for providing and delivering medications; and (4) simplified glucose drug regimens should be used when possible. However, challenges remain. Adherence to prescribed regimens is difficult because of disrupted lifestyles, ongoing attacks, long periods spent in shelters and severe stress. There is still limited access to care, including diabetes care, in areas near the zones of heavy fighting. Many new cases of diabetes and its complications are anticipated, and high rates of diabetes-related foot ulcers and related amputations have been reported [47]. Optimising diabetes care and health outcomes will be a major post-war task.

### Case study: the Tigray war

Similar diabetes care-related challenges and solutions were experienced during the Tigray war in Ethiopia (3 November 2020–3 November 2022). Tigray is one of 11 regions of Ethiopia, with an estimated population in 2020 of 7.3 million [48]. The region, located in the north of the country, borders the Afar and Amhara regions, Sudan and Eritrea.

The Tigray war was fought between the Ethiopian federal government and Eritrea allied forces and the Tigray Defence Forces. The war resulted in a full-scale humanitarian catastrophe, with >70,000 refugees fleeing from Western Tigray to Sudan, >2 million displaced people sheltering in different parts of Tigray and approximately 518,000 civilian deaths [49]. During this period, Tigray was under siege and humanitarian blockade, with critical shortages of food and medicines leading to mass starvation and the complete collapse of the healthcare system.

**Fig. 4:**
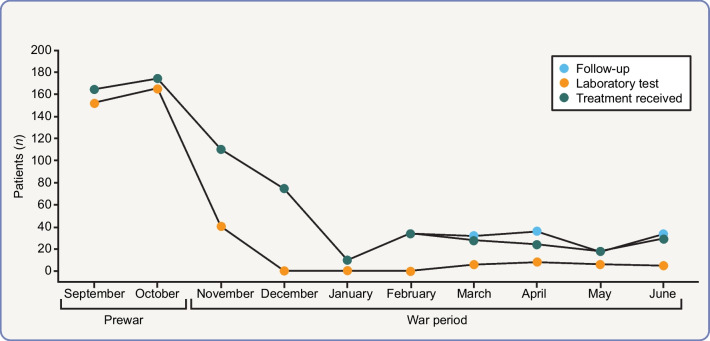
Changes in the use of services by individuals with type 1 diabetes before and during the Tigray war (September 2020–June 2021). Reproduced from Gebrehiwet et al [48]. This figure is available as part of a downloadable slideset

Médecins Sans Frontières (Doctors Without Borders [https://www.msf.org]) teams in Tigray documented evidence of widespread, deliberate and targeted attacks on health facilities. Before the war, Tigray had >720 health posts, 224 health centres, 24 primary hospitals, 14 general hospitals and two tertiary specialised hospitals. However, of 106 facilities assessed between mid-December 2020 and early March 2021, 87% were no longer functioning or fully functioning [50]. The war led to the complete breakdown of healthcare delivery, with no health posts, only 3.6% of health centres and 13.5% of hospitals being fully functional in 2021 [51]. As expected, this had a major negative impact on chronic disease management, with only 21% of people using healthcare services from November 2020 to June 2021 compared with prewar data (September–October 2020) [48] (Fig. 4).

In Tigray as a whole, although underestimates owing to underdiagnosis and limited access to diabetes care, there were 6726 people with type 1 diabetes and 17,627 with type 2 diabetes receiving regular follow-up at general and primary hospitals pre war in November 2020 [52]. About 80% of essential medications were available before the war, which plummeted to <20% by the start of 2022; similarly, the availability of routine laboratory tests, including blood glucose and HbA_1c_ testing, dropped from 94% to <50% by the start of 2022 [53]. Insulin supplies declined sharply during the war and completely ran out by July 2021, resulting in avoidable deaths; doctors were forced to use expired drugs and individuals with diabetic ketoacidosis were treated with oral hydration because of a lack of i.v. fluids; and increased levels of malnutrition also complicated diabetes management, as individuals with diabetes had reduced resilience to fight communicable diseases [52]. In addition, deaths of dialysis patients doubled because of interruptions to dialysis sessions [54], and healthcare professionals worked without pay for 17 months while facing severe food shortages [55]. The IDF publicised this humanitarian crisis in January 2022 [52], forcing the Ethiopian government to send limited supplies of insulin to Tigray [56, 57]. Although the war ended after the Cessation of Hostilities Agreement in 2022, healthcare services in Tigray have not yet recovered, and access to insulin and other critical medications still remains limited. Unfortunately, many healthcare professionals left their posts, seeking employment in other countries, further exacerbating chronic disease care [58]. The war has had a major psychological impact on the public, including people with diabetes, with increasing levels of anxiety and depression [57, 58]. The Tigray war demonstrated that it takes only a few weeks for diabetes care to collapse and many years for it to recover. Almost all sections of society, including medical doctors, were seriously affected by the lack of insulin, with many deaths occurring, especially among children [57, 59]. The Tigray war highlights how strategies for rapid insulin access in areas hit by war and disasters need to be developed and ideally agreed at the United Nations and by all countries.

## Role of the IDF in diabetes-related disaster management

While many organisations (e.g. Direct Relief, Red Cross, UN, WHO and Doctors Without Borders) perform disaster-related work, the IDF is highlighted here as it has a diabetes focus and has developed comprehensive resources, which are freely available on various websites (see the following section). The IDF is a non-profit umbrella organisation of >240 national diabetes associations in 160 countries. One of its major humanitarian activities is to prioritise the needs of people with diabetes affected by natural or man-made disasters. The IDF has partnered with Direct Relief since 2009 to provide diabetes medications and monitoring to vulnerable populations during humanitarian emergencies. In 2019, the IDF and Direct Relief began a pilot collaboration to encourage healthcare manufacturers to donate diabetes-related medications and supplies for diabetes care during disasters. This partnership has resulted in >5 million oral tablets, tens of thousands of vials of insulin and diabetes consumables being distributed to countries on four continents in collaboration with relevant member associations. Recently, emergency relief has been provided to Ukraine following the Russian invasion in 2022 and to Turkey/Syria in the aftermath of the devastating earthquake in 2023. Over the last year, the IDF, through its member associations in Palestine, Lebanon and especially Egypt, has tried to assist in the catastrophic situation facing those living with diabetes in the Gaza Strip. Other aid organisations such as Insulin For Life also assist following disasters, particularly in the very early days and weeks and often continuing long term; for example, Insulin For Life is still sending insulin and related supplies to Ukraine.

### The IDF-WPR and its disaster-related challenges and resources

The IDF-WPR, the most populous IDF region, with approximately 163 million people living with diabetes in 2021 [2], includes a range of countries from economically advantaged to less advantaged, with many having additional challenges related to geography that can affect travel, supply chains and communications during disasters. The IDF-WPR is frequently challenged by disasters, most commonly natural disasters such as bushfires (wildfires), earthquakes, tsunamis, floods and COVID-19 [13].

As already highlighted in this review, detailed and timely preparation lessens the negative impacts of disasters on people with diabetes. In this regard, in 2022, the IDF-WPR released the second edition of its Diabetes Care and Disasters guidelines [13, 14], which are available for free on the IDF website (https://idf.org/news/new-edition-of-diabetes-care-and-disasters-idf-western-pacific-region-manual/) and at https://www.ajenkinsdiabetes.org/html/pages/diabetes-in-disasters. The latter website also includes free diabetes and disaster-related guidelines from Australia, China, Japan and South Korea and articles describing how the IDF-WPR responded to the COVID-19 pandemic. Local guidelines are the best guidelines, and groups are welcome to adopt and adapt the IDF-WPR Diabetes Care and Disasters guidelines.

The IDF-WPR Diabetes Care and Disaster guidelines (2nd edition) include expanded checklists for people with diabetes, including emergency kit contents; recipes for home-made hand and surface sanitisers and water purification and rehydration solutions; insulin switching charts; information on insulin storage and stability when not refrigerated; information on safe sharps reuse and disposal by individuals; blood and interstitial fluid glucose levels and HbA_1c_ unit conversion charts; guidelines for the emergency treatment of diabetic ketoacidosis; information on a basic baby delivery kit; advice for first responders; guidelines for palliative care; human rights aspects of healthcare during a disaster; and who and how to ask for help [13, 14]. A slide presentation to facilitate dialogue is also available.

## Conclusions

**Fig. 5:**
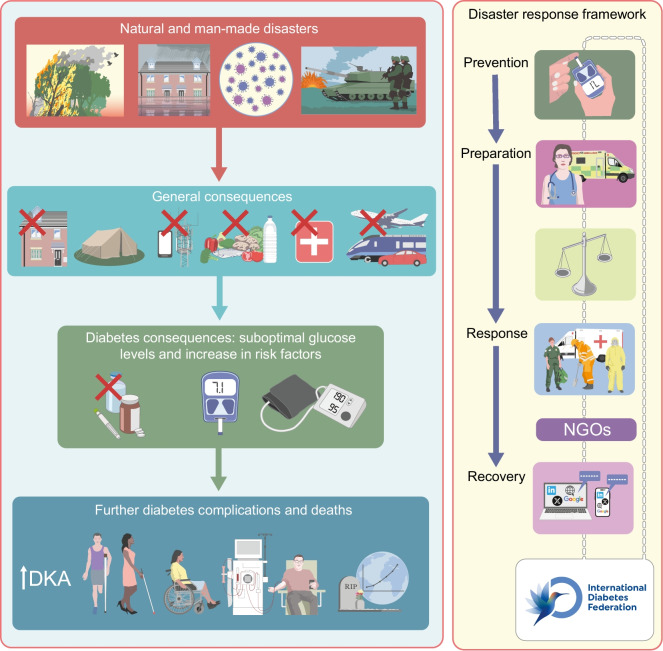
Schematic summarising the potential consequences of natural and man-made disasters and the resources available to prevent, prepare for, respond to and recover from a disaster. Linkages between many individuals and organisations may assist as part of the disaster response framework. DKA, diabetic ketoacidosis; NGO, non-governmental organisation. This figure is available as part of a downloadable slideset

This review has focused on the management of diabetes and support for people living with diabetes in countries across the world affected by natural or man-made disasters. Examples of the problems faced and potential solutions from recent disasters are discussed and are summarised in Fig. 5. The IDF-WPR guidelines on diabetes care and disasters focus on the important principles of prevention, preparedness, response and recovery, which all those looking after people with diabetes should be aware of wherever they are located in the world. Sadly, both natural and man-made disasters appear to be increasing in incidence; thus, preparedness and united action is of vital importance to save people living with diabetes from avoidable morbidity and premature death due to lack of essential medications. Post-disaster assessments and sharing of experiences and resources are recommended.

## Supplementary Information

Below is the link to the electronic supplementary material.

Slideset of figures (PPTX 630 KB)

## Abbreviations

IDF: International Diabetes Federation
IDF-WPR: International Diabetes Federation-Western Pacific Region

## Authors’ relationships and activities

AJMB is the immediate Past-President of the IDF (2019–2022), Past-President of the EASD (2011–2015) and President of the Worldwide Initiative for Diabetes Education (2022–2025). He serves on the advisory boards for Nevro, AOT, Viatris and Wörwag. AJJ is the IDF-WPR Chair Elect, editor/author of the IDF-WPR Diabetes Care and Disasters guidelines (2nd edition), Chair of the IDF Disaster Committee and President of Insulin For Life Global; is a board member at Insulin for Life Australia; and collaborates with Life For a Child. She serves on the advisory boards for Abbott and GSK and lectures for CSL Seqirus. She has received research grants from Medtronic, Insulet, Ypsomed and Abbott/Viatris. BrM is the immediate Past-President of the Research Society for the Study of Diabetes in India (RSSDI) and Honorary Secretary of the AIAARO (Indian Obesity Society) (2023–2025). He serves on the advisory boards for or has received speaking fees from AstraZeneca, Boehringer Ingelheim, Eli Lilly, Novo Nordisk, Sanofi, Roche, LifeScan, Alembic, Alkem, Cipla, Torrent and USV. BoM is the President of the Ukrainian Diabetology Association. He has received advisory board and speaking fees from AstraZeneca, Bayer, Boehringer Ingelheim, Novartis, Novo Nordisk, Sanofi, Servier and Wörwag Pharma. ST has received consulting or speaker’s honoraria from Viatris, Nevro, Novo Nordisk, Merck, Grunenthal, P&G Health, Bayer, Wörwag Pharma, Angelini, Confo Therapeutics, AstraZeneca, NeuroPn and Merz. The authors declare that there are no other relationships or activities that might bias, or be perceived to bias, their work.
