# Supplementary material for: Diabetes and natural and man-made disasters: prevention, preparation, response and recovery
Source: Diabetologia. 2025 Apr 15;68(11):2465–78. doi: 10.1007/s00125-025-06406-6 (PMC12534289; doi:10.1007/s00125-025-06406-6)
Supplement: Supplementary file 1 — Slideset of figures (PPTX 630 KB) [file 125_2025_6406_MOESM1_ESM.pptx]

## Slide 1
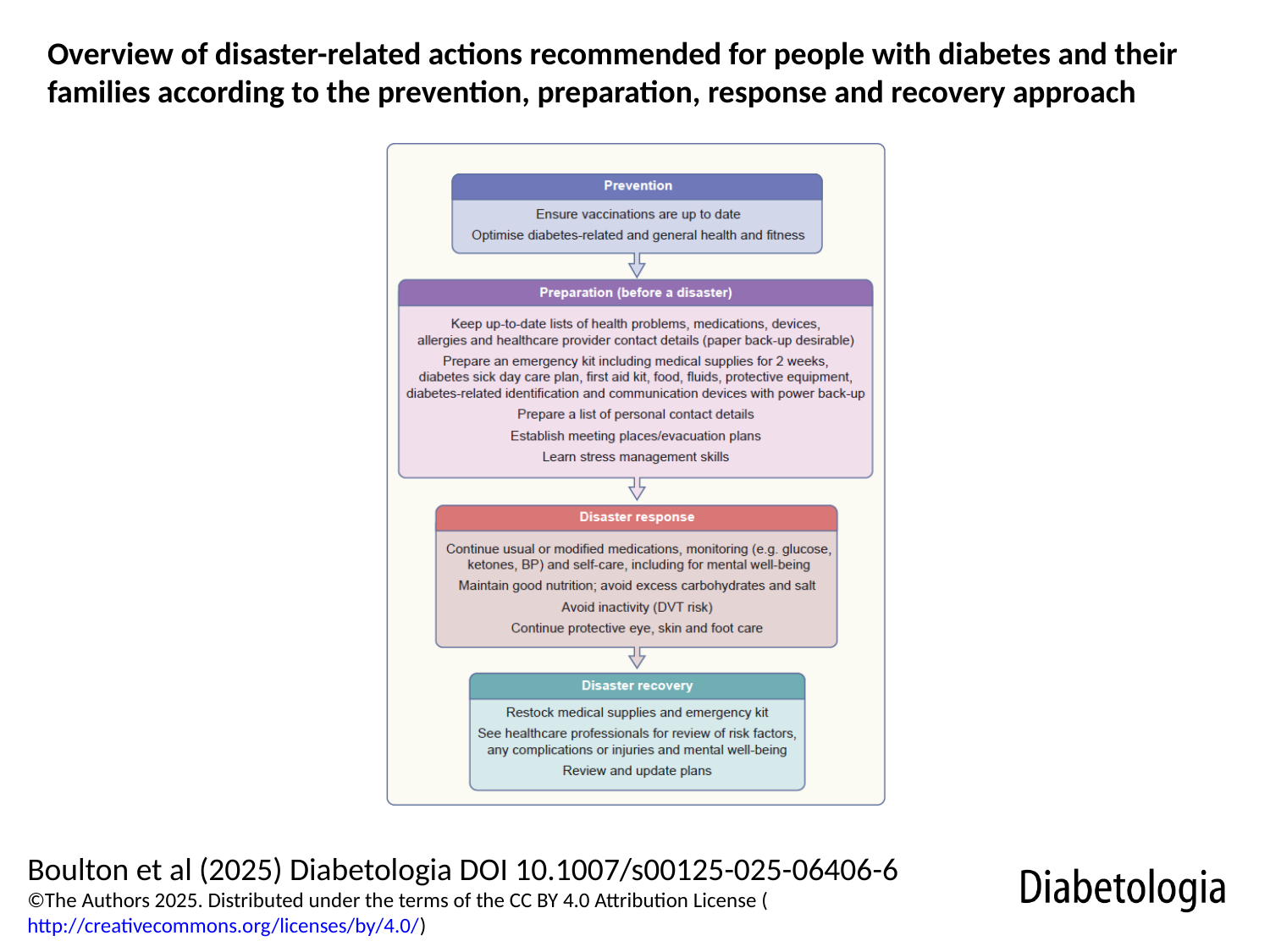

Overview of disaster-related actions recommended for people with diabetes and their families according to the prevention, preparation, response and recovery approach
Boulton et al (2025) Diabetologia DOI 10.1007/s00125-025-06406-6
©The Authors 2025. Distributed under the terms of the CC BY 4.0 Attribution License (http://creativecommons.org/licenses/by/4.0/)

## Slide 2
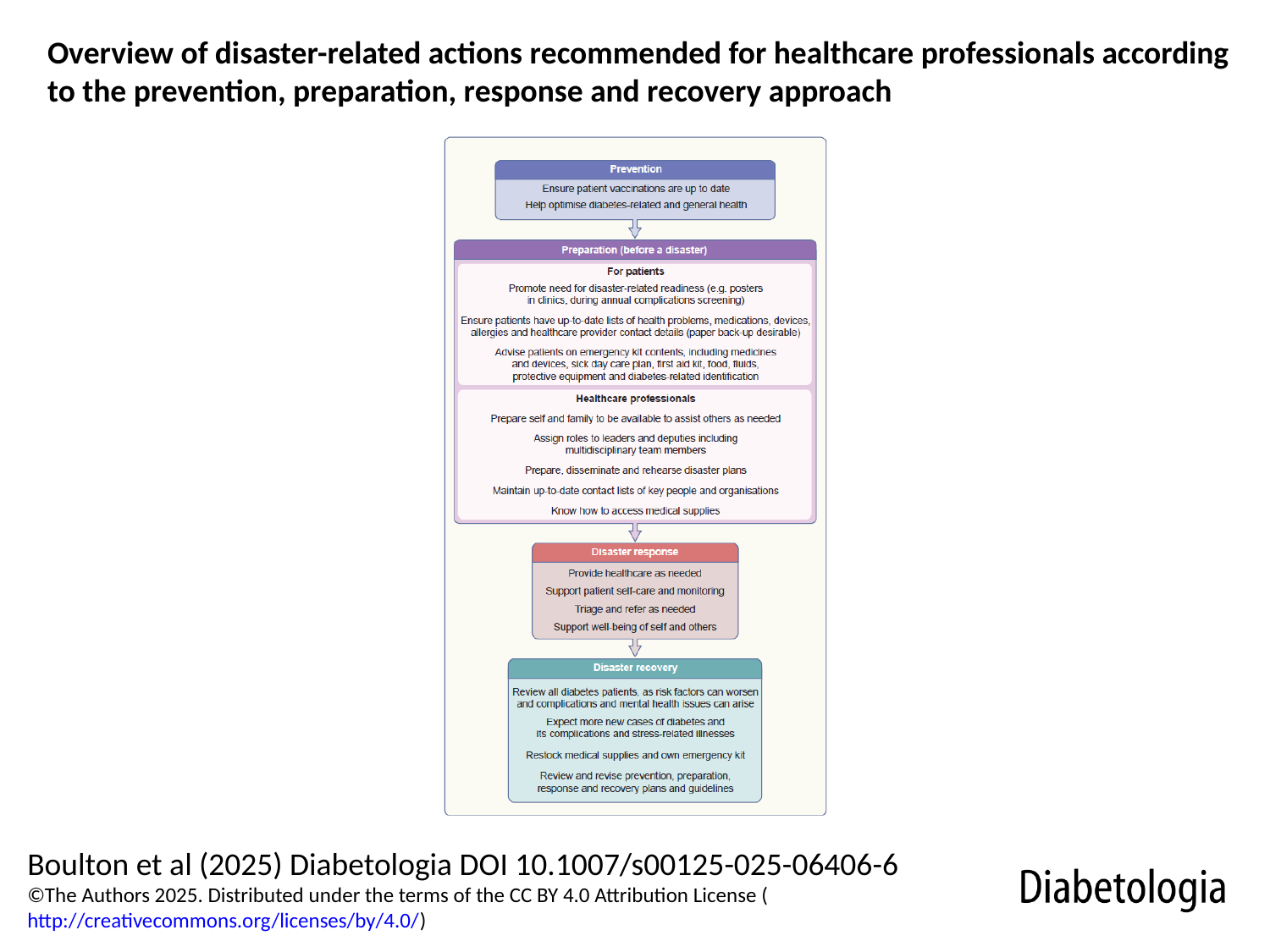

Overview of disaster-related actions recommended for healthcare professionals according to the prevention, preparation, response and recovery approach
Boulton et al (2025) Diabetologia DOI 10.1007/s00125-025-06406-6
©The Authors 2025. Distributed under the terms of the CC BY 4.0 Attribution License (http://creativecommons.org/licenses/by/4.0/)

## Slide 3
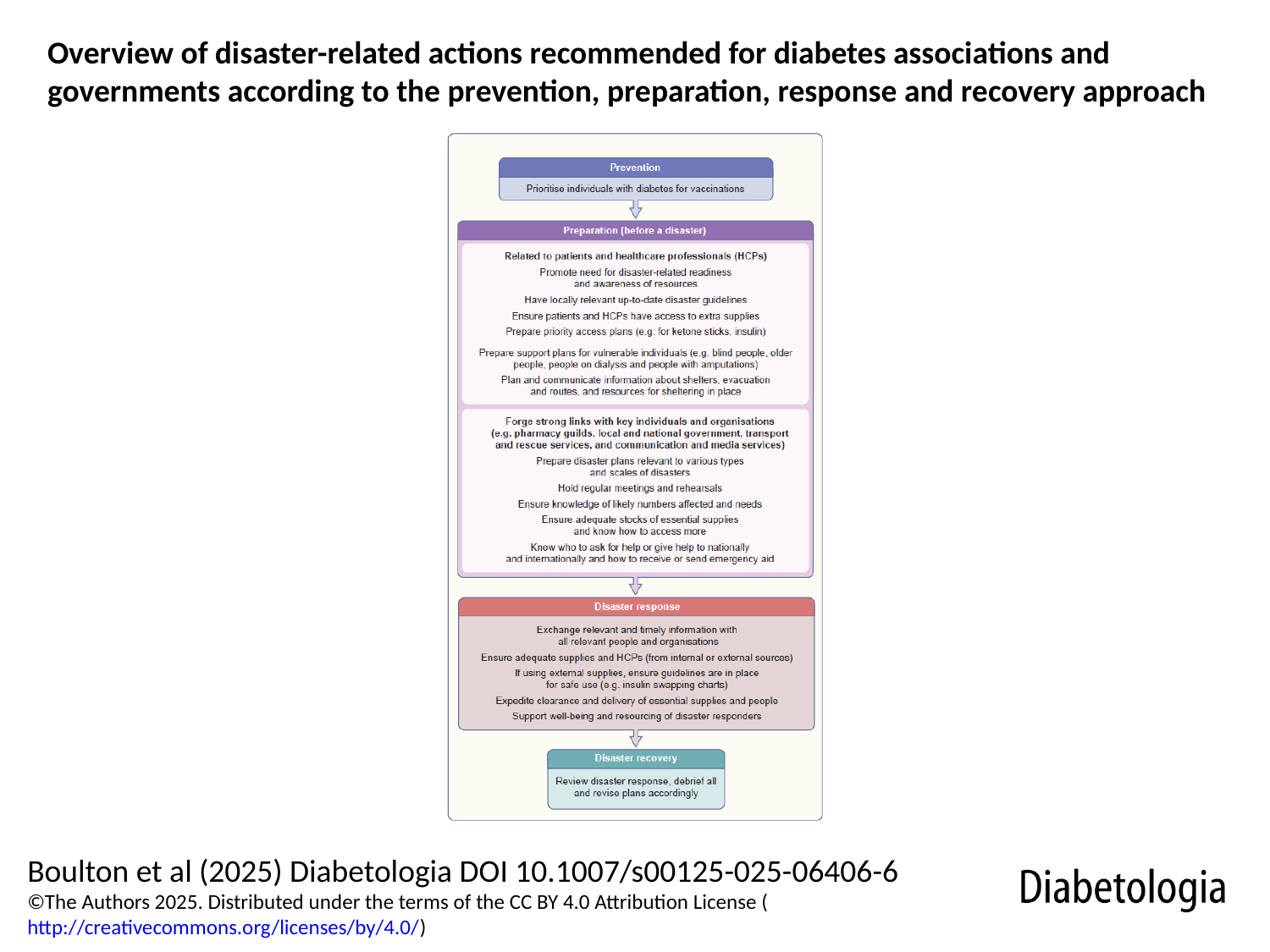

Overview of disaster-related actions recommended for diabetes associations and governments according to the prevention, preparation, response and recovery approach
Boulton et al (2025) Diabetologia DOI 10.1007/s00125-025-06406-6
©The Authors 2025. Distributed under the terms of the CC BY 4.0 Attribution License (http://creativecommons.org/licenses/by/4.0/)

## Slide 4
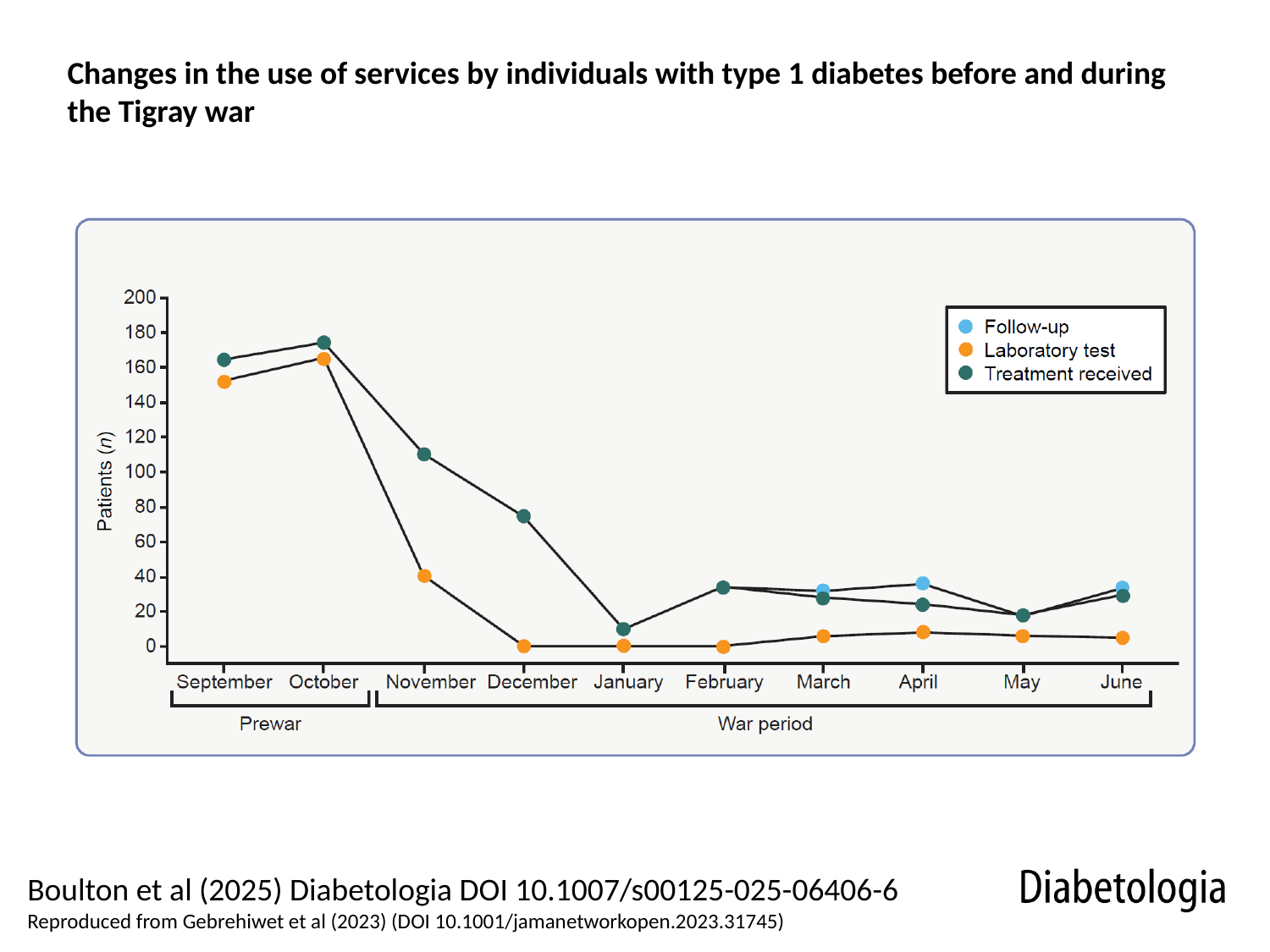

Changes in the use of services by individuals with type 1 diabetes before and during the Tigray war
Boulton et al (2025) Diabetologia DOI 10.1007/s00125-025-06406-6
Reproduced from Gebrehiwet et al (2023) (DOI 10.1001/jamanetworkopen.2023.31745)

## Slide 5
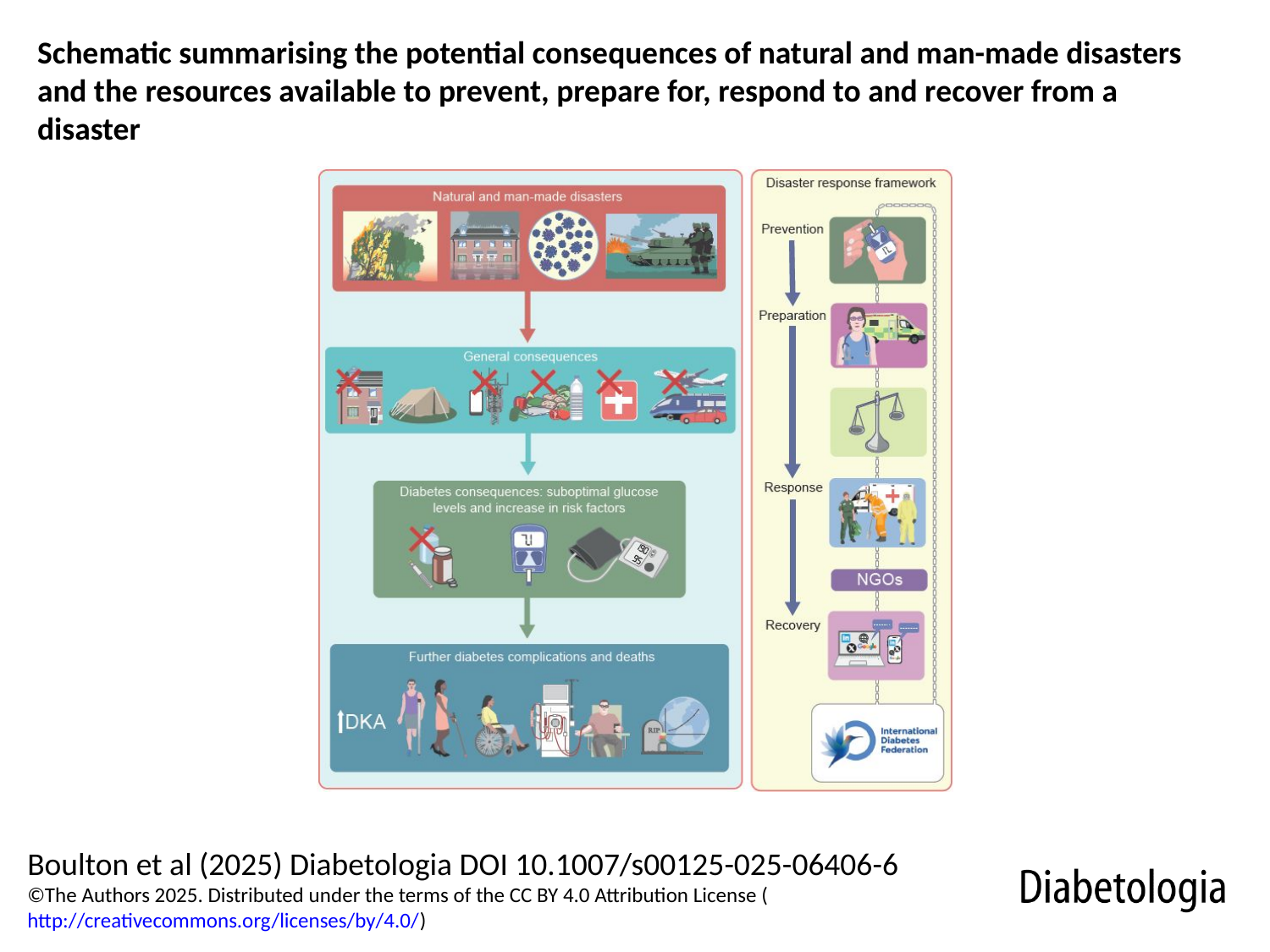

Schematic summarising the potential consequences of natural and man-made disasters and the resources available to prevent, prepare for, respond to and recover from a disaster
Boulton et al (2025) Diabetologia DOI 10.1007/s00125-025-06406-6
©The Authors 2025. Distributed under the terms of the CC BY 4.0 Attribution License (http://creativecommons.org/licenses/by/4.0/)
